# Diagnostic algorithm for Primary Ciliary Dyskinesia: recommendations of the French National Centre for Rare Respiratory Diseases

**DOI:** 10.1186/2046-2530-1-S1-P5

**Published:** 2012-11-16

**Authors:** S Blanchon, L Bassinet, N Beydon, A Clément, E Escudier, JF Papon, A Tamalet

**Affiliations:** 1 Dept Pediatric Pulmonology, Hôpital Trousseau,France; 2 Dept Pulmonology, Centre Hospitalier Intercommunal, Créteil, France; 3 Dept Lung Physiology, Hôpital Trousseau, France; 4 Dept Medical Genetics and Embryology, Hôpital Trousseau, France; 5 ENT Department, Hôpital Henri Mondor, Créteil, France

## Background

Primary Ciliary Dyskinesia (PCD) is a inherited disorder characterized by abnormal ciliary structure/function, responsible for impaired muco-ciliary transport and infertility. The diagnosis is often delayed for several years though PCD is a rare (~1/20,000) and heterogeneous disease. We elaborated recommendations aiming at improving the quality and efficacy of PCD diagnosis.

## Methods

A multicentric working group, created in 2009 within the French National Centre for Rare Respiratory Diseases, elaborated precise and practical algorithm for PCD diagnosis, based on their experience and the recent literature. It includes adults and paediatric physicians, biologists and technicians involved in PCD, dealing since many years with the difficulties to confirm this diagnosis.

## Results

PCD diagnosis should be clinically suggested by general features (recurrent upper and lower airways infections early in life, laterality defects, infertility, familial consanguinity or PCD history), concurrent with ENT (nasal polyposis, chronic rhinosinusitis, otitis media) and pulmonary symptoms (chronic bronchitis, bronchiectasis). After exclusion of overlapping pathologies (cystic fibrosis, immunodeficiency), a ciliary dysfunction (assessed by nasal nitric oxide and/or ciliary beat frequency analysis) is a necessary criteria to select patients requiring ultrastructural analysis by electron microscopy, our gold standard for diagnosis confirmation. High-speed-video-microscopy could be helpful in case of discrepancy between clinical features, functional and structural ciliary analysis.

## Conclusion

The French National Centre reminds the features of PCD, and describes a step by step algorithm for diagnosis. Based on these recommendations, a more accurate approach in patients suspected for PCD will improve the quality and efficacy of investigations; reducing the delay to diagnosis.

